# Change-Point Analysis of Eye Movement Characteristics for Female Drivers in Anxiety

**DOI:** 10.3390/ijerph16071236

**Published:** 2019-04-07

**Authors:** Yongqing Guo, Xiaoyuan Wang, Qing Xu, Feifei Liu, Yaqi Liu, Yuanyuan Xia

**Affiliations:** 1 School of Transportation and Vehicle Engineering, Shandong University of Technology, Zibo 255049, China; yongqing.guo@sdut.edu.cn (Y.G.); liufeifei19911218@163.com (F.L.); liuyaqi518@126.com (Y.L.); 2 College of Electromechanical Engineering, Qingdao University of Science & Technology, Qingdao 266000, China; xyymjq@aliyun.com; 3 Joint Laboratory for Internet of Vehicles, Ministry of Education—China Mobile Communications Corporation, Tsinghua University, Beijing 100048, China; 4 Department of Automotive Engineering, Tsinghua University, Beijing 100084, China; qingxu@tsinghua.edu.cn

**Keywords:** driving anxiety, eye movement, change-point analysis, least squares method

## Abstract

Driver hazard perception is highly related to involvement in traffic accidents, and vision is the most important sense with which we perceive risk. Therefore, it is of great significance to explore the characteristics of drivers’ eye movements to promote road safety. This study focuses on analyzing the changes of drivers’ eye-movement characteristics in anxiety. We used various materials to induce drivers’ anxiety, and then conducted the real driving experiments and driving simulations to collect drivers’ eye-movement data. Then, we compared the differences between calm and anxiety on drivers’ eye-movement characteristics, in order to extract the key eye-movement features. The least squares method of change point analysis was carried out to detect the time and locations of sudden changes in eye movement characteristics. The results show that the least squares method is effective for identifying eye-movement changes of female drivers in anxiety. It was also found that changes in road environments could cause a significant increase in fixation count and fixation duration for female drivers, such as in scenes with traffic accidents or sharp curves. The findings of this study can be used to recognize unexpected events in road environment and improve the geometric design of curved roads. This study can also be used to develop active driving warning systems and intelligent human–machine interactions in vehicles. This study would be of great theoretical significance and application value for improving road traffic safety.

## 1. Introduction

During driving, drivers usually receive more than 80% of traffic environment information through their vision [1,2]. The sudden changes of driver’s eye movement are closely associated with road traffic crashes. Therefore, it is necessary to investigate drivers’ eye movement changes for improving road safety.

Transportation scholars have explored drivers’ visual search modes in various types of roads [3,4,5,6,7,8] (e.g., mountain roads, roundabouts, overpasses, curved roads, and grassland roads) and different driving environments [9,10,11,12,13,14,15] (e.g., road construction, tunnel entrances, tunnel lighting environments, and traffic signs and billboards). It was found that drivers’ eye movements change with the driving environment.

Previous research suggests that there are relationships among driving anxiety, visual search mode, and driving behavior. Taylor et al. [16] found that drivers with high anxiety are more likely to make mistakes during driving, based on the results of a questionnaire survey. Pourabdian [17] used the Manchester Driving Behavior Questionnaire and the Spielberg State–Trait Anxiety Scale to analyze the relationship between trait anxiety level and inappropriate driving behavior, and found that trait anxiety level is prone to causing driver distraction and forgetfulness. Wester et al. [18] found that with a high level of anxiety, drivers have an increased fixation frequency in the central area and a decreased fixation frequency on both sides (narrowed attention). Anxiety can narrow driver attention, which results in ignoring important peripheral information and increased driving risk [19].

There have been a number of studies showing driver’s eye movement characteristics. Hills et al. [20,21] found that driver’s eye movements change with the level of driving experience. It is not easy for novice drivers to apply appropriate eye-movement patterns to match the hazardousness of the road. Foy et al. [22] studied the relationship between driver’s mental workload and eye movement characteristics. It was found that an increased mental workload leads to wider visual search breadth and longer gaze duration. Le et al. [23] combined a vestibulo-ocular reflex model with a visual-kinematic reflex model to explore the effects of psychological load on driver’s eye movement. Based on the findings, they developed a system to monitor drivers’ visual behavior. Bakhit et al. [24] analyzed the relationship between eye scanning behavior and different levels of driving tasks, and also developed two measures for distracted driving.

Drivers’ eye-movement analysis has received increasing attention in traffic safety research. Li et al. [25] analyzed drivers’ eye-movement characteristics in accident-prone areas, focusing on drivers’ cognitive workload and fixation point distribution. The findings of the study were used to provide effective strategies to improve road safety. He et al. [11] analyzed the effects of highway tunnel lighting environment on driving safety, using drivers’ eye movement parameters. Hills et al. [20] explored the vertical eye-movement carryover from one task to a second task, and found that it is one potentially distracting effect on the safety of novice drivers. Oviedo-Trespalacios et al. [26] conducted a systematic literature review to identify the impacts of roadside advertising signs on driver behavior and road safety. The findings suggested that roadside advertising can increase crash risk, particularly when signs that are frequently changed. Vignali et al. [27] examined pedestrians’ first-fixation distance of the crosswalk to optimize the crosswalk design from a perspective of accident prevention. Costa et al. [28] analyzed drivers’ first-fixation distance and fixation duration distributions to vertical road signs in order to identify the fixation time for the driver to correctly identify a road sign. Lantieri et al. [29] explored the effect of gateways to reduce the amount of distraction, through analyzing drivers’ eye movement data.

In summary, most research on drivers’ eye movements has focused on exploring drivers’ eye movement characteristics, as well as the relationship between visual search mode and driving emotion. Few studies have attempted to investigate the change points of eye movement characteristics with mathematical models and algorithms. This paper will use the least squares of change-point estimation to detect and analyze eye movement changes of female drivers in anxiety.

## 2. Materials and Methods

The change-point method is a powerful tool to detect whether any changes have occurred [30,31,32,33,34]. It can determine the number of changes and estimate the time and location of each change. It is also capable of detecting small changes, and is preferable particularly when dealing with large data sets. Generally, a change-point analysis characterizes the changes accurately, controls the error rate strongly, is robust to outliers, and is simple to use. Wang et al. have applied the change-point models to analyze traffic flow theory, including the mean change-point model [35], the non-linear probability change-point model [36], the least squares method, and the local comparison method [37]. Therefore, the change point method was used for detecting drivers’ eye movement changes in this study.

### 2.1. Formulation of the Change-Point Model

**Figure 1:**
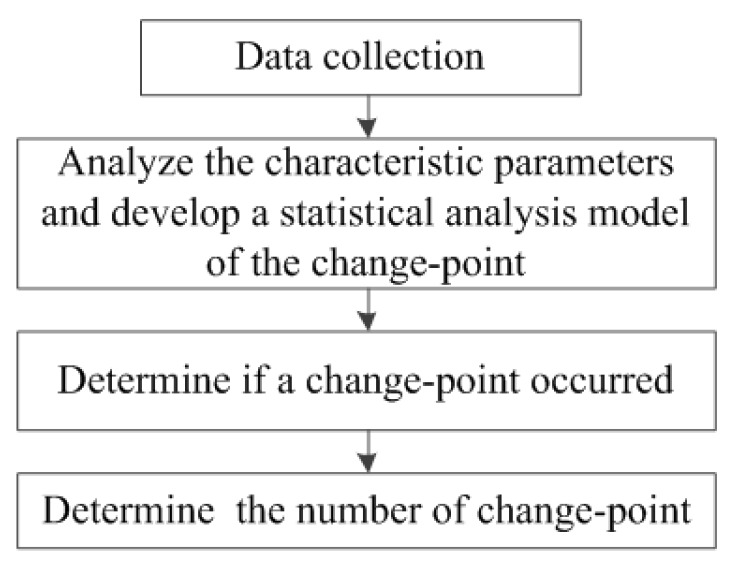
Change-point analysis process.

In a change-point problem, there are a series of observations, which are mostly arranged according to the time of their occurrence. At an unknown moment, if the distribution of samples or their numerical characteristics suddenly change, the moment is probably the point of change. Assuming that *X_i_*(1 ≤ i ≤ n) is a sample from the matrix *X*, we want to study the samples in order to determine whether there is a significant change in the matrix, where the change occurs, and how large the change is. Assuming that only one change occurs, the sample *X_i_* before and after the change-point follows a normal distribution with variance $\sigma^{2}$, but the expected value of the distribution varies. The location of the change is denoted as *m* (unknown). Therefore, the issue of “whether there is a change” can be considered to a hypothesis test question under the premise that “$X_{1},\;\cdots {,\;X}_{n}$” are mutually independent, and follow a normal distribution with variance $\sigma^{2}$:

Null hypothesis H: $EX_{1}=EX_{2}=\cdots =EX_{100}$ (no change-point);

Alternative hypothesis K: for *m*, 1 ≤ m ≤ 100, and $a_{1}\neq a_{2}$

(1) $${\mathrm{EX}}_{1}=\cdots ={\mathrm{EX}}_{m-1}=a_{1},\;{\mathrm{EX}}_{m}=\cdots ={\mathrm{EX}}_{100}=a_{2}\;(\mathrm{change}\text{-}\mathrm{point}\;m)$$

where *m* (unknown) is called a change-point, that is, “the point in time when a sudden change occurs”. If null hypothesis is rejected, the position of change-point m and the change range (jump degree) $a_{2}-a_{1}$ will be determined. The analysis process of change point is shown in Figure 1.

### 2.2. Least Square Method of the Change-Point Analysis

#### 2.2.1. Mean Change-Point Model with Known Number of Change Points

Firstly, we determine a natural number *q*, and the number of change points should be less than *q*. Compared to the sample size *n*, *q* is a rather small number. The discrete data model of the mean change-point problem is:

(2) $$\{\begin{array}{c} X_{i}=a_{i}+e_{i},i=1,\cdots ,n\\ a_{1}=\cdots =a_{m_{1}-1}=b_{1}\\ a_{m_{1}}=\cdots =a_{m_{2}-1}=b_{2}\\ \cdots \\ a_{m_{q}}=\cdots =a_{n}=b_{q+1} \end{array}$$

where *X_i_* stands for the sample (as in Equation (1)), *n* represents the number of observation positions, and $1{\;<\;m}_{1}{\;<\;m}_{2}{\;<\;\cdots \;<\;m}_{q}\;\leq \;n$. If $b_{j+1}\;\neq {\;b}_{j}$, mi is the change point. Random error $e_{i}$ (*i* = 1, …, *n*) is assumed to be independent, with equal variance $\sigma^{2}$ and expected value 0.

#### 2.2.2. Least Square Method of the Change-Point Search

The sum of the squared differences between observed and theoretical values is chosen as objective function, in order to obtain the minimum value as the point estimate of the population parameter.

The objective function is established as follows:

(3) $${T\;=\;T(m}_{1},\cdots {,m}_{q}{,b}_{1},\cdots {,b}_{q+1})\;=\sum_{j=1}^{q+1}\sum_{{i=m}_{j-1}}^{m_{j}-1}{{(x}_{i}\;{-\;b}_{j})}^{2}$$

Here, $m_{0}{\;=\;1,m}_{q+1}\;=\;n+1$. $m_{1},\cdots ,m_{q}$ remain unchanged; finding the minimum value of T, we obtain:

(4) $$b_{j}{\;=\;Y}_{j}{\;=X}_{m_{j}-1}\;+\;\cdots {\;+\;X}_{m_{j-1}}/{(m}_{j}{\;-\;m}_{j-1})$$

Equation (3) reaches the minimum. By substituting Equation (4) into Equation (3), we get:

(5) $${T(m}_{1},\cdots {,m}_{q})\;=\sum_{j=1}^{q+1}\sum_{{i=m}_{j-1}}^{m_{j}-1}{{(X}_{i}{\;-\;Y}_{j})}^{2}$$

In the range of ${1\;<\;m}_{1}\;<\;\cdots {\;<\;m}_{q}\;\leq \;n$, Equation (5) is adjusted iteratively to reach the minimum. The steps are:

(1) Set a set of initial values $m_{1},\;\cdots {,\;m}_{q}$ (${1\;<\;m}_{1}\;<\;\cdots {\;<\;m}_{q}\;\leq \;n$);
(2) Select the first two terms of Equation (5); the sum is calculated by:
  (6) $$W=\overset{m_{1}-1}{\underset{i=1}{\sum }}{\left(X_{i}-Y_{1}\right)}^{2}+\overset{m_{2}-1}{\underset{i=m_{1}}{\sum }}{\left(X_{i}-Y_{2}\right)}^{2}$$
  where *Y_1_* and *Y_2_* are calculated by Equation (4) based on the initial values $m_{1},\;\cdots {,\;m}_{q}$. *m*_2_ remains unchanged, and *m*_1_ is adjusted in a range of 1 < *m*_1_ < *m*_2_ to minimize W; that is, *n* = *m*_2_, *q* = 1. W reaches the minimum value *m*_1,_ denoted as $m_{1}^{\prime }$.
(3) The sum of the second and third terms of Equation (5) is determined by substituting $m_{1}^{\prime }$ into *m*_1_; we obtain:
  (7) $$W=\overset{m_{2}-1}{\underset{i=m_{1}^{\prime }}{\sum }}{\left(X_{i}-Y_{2}\right)}^{2}+\overset{m_{3}-1}{\underset{i=m_{2}}{\sum }}{\left(X_{i}-Y_{3}\right)}^{2}$$
  where *Y_2_* and *Y_3_* are calculated by Equation (4). However, *m*_1_ is replaced by $m_{1}^{\prime }$, and $m_{1}^{\prime }$ and *m*_3_ remain unchanged, while $m_{2}$ is adjusted within $m_{1}^{\prime }<m_{2}<m_{3}$ to minimize W. W reaches the minimum value *m*_2_, denoted as $m_{2}^{\prime }$.
(4) $m_{2}^{\prime }$ and *m*_4_ remain unchanged, and *m*_3_ is adjusted to get $m_{3}^{\prime }$. Iteratively, a set of new values $m_{1}^{\prime \prime }$, …, $m_{q}^{\prime \prime }$ are obtained. Take them as initial values, go back to the first step to get another set of new values $m_{1}^{\prime \prime }$, …, $m_{q}^{\prime \prime }$, and then go back to the first step. This iterative process continues until there is no adjustment needed (the new value exactly equals to the previous one). The final value, which is denoted as ${\hat{m}}_{1}\cdots {\hat{m}}_{q}$, can be used as an estimate of the change-point $m_{1},\;\cdots {,\;m}_{q}$. The minimum value of T in Equation (3) is T($m_{1},\;\cdots {,\;m}_{q}$), denoted as $T_{q}$.

#### 2.2.3. Estimating the Number of Change-Points

When analyzing the change-point problems, we follow the following steps: the first step is to detect if there is a change-point or not. If the null hypothesis (no change-point) is accepted, it means that there will be no change-point (the test method is presented below); if it is rejected, at most, *q* change points are allowed to be proposed. The following methods are used: put *q* = *k* in Equation (3) and determine the minimum value *T_k_* of *T*, that is *T*_1_, ⋯, *T_k_*. We can see *T*_1_ ≥ *T*_2_ ≥ ⋯ ≥ *T_k_*. Considering the structure of Equation (3) and the minimum value of formula $\sum {\left(a_{i}-b\right)}^{2}$ at b=a, if there are only *k* change points, *T_k_* is not much greater than *T_K_*_+1_. Otherwise, if there are more than *k*, *T_k_* is significantly higher than *T_K_*_+1_ (the jump size of parameter mean at the change-point should be considered). The following empirical rules are often used to study practical problems: if the values decrease dramatically from *T*_1_ to *T_k_* and keep flattening after that, the estimated value of the change-point is k. After determining *k*, q in Equation (3) is substituted by *k*, and the values of ${\hat{m}}_{1}\cdots {\hat{m}}_{q}$ are obtained by minimizing *T* as the estimate of the change-point position.

The minimum value of $T_{k}/T_{q}$ (in Equation (5)) can be considered as $T_{q}$, which is more than 1. A number slightly larger than 1 can be set, such as 1.1, making $T_{k}/T_{q}\;\geq 1.1$ the change-point estimate.

#### 2.2.4. Hypothesis Test Problem of Change-Point

Regarding the problem of “whether there is a change-point or not”, the hypothesis testing procedure is shown below. The null hypothesis H denotes a hypothesis in which there is no change-point. That is, in Equation (2), $b_{1}\;=\;\cdots {\;=\;b}_{q+1}$. The variances of samples $X_{1},\cdots ,X_{n}$ (not divided by degrees of freedom, the same below) are given by:

(8) $$S\;=\sum_{i=1}^{n}{\left(X_{j}-\bar{X}\right)}^{2}\;(\bar{X}=\sum_{i=1}^{n}X_{i}/n)$$

Divide samples $X_{1},\cdots ,X_{n}$ into two segments, $X_{1},\cdots ,X_{i-1}$ and $X_{i},\cdots ,X_{n}$, calculate their variances respectively, and put them together:

(9) $$S_{i}=\;\overset{i-1}{\underset{j=1}{\sum }}{{(X}_{j}\;-{\bar{\;X}}_{i1})}^{2}+\overset{n}{\underset{j=i}{\sum }}{{(X}_{j}\;-{\bar{\;X}}_{i2})}^{2}(2\;\leq \;i\;\leq \;n)$$

$\;X_{i1}$ and $X_{i2}$ are the arithmetic means of the segments $x_{1},\;\cdots {,\;x}_{i-1}$ and $x_{i},\;\cdots {,\;x}_{n}$, respectively. To simplify, use the identity below:

(10) $$S=S_{i}+n^{-1}(i-1)(n-i+1){\left({\bar{X}}_{i1}-{\bar{X}}_{i2}\right)}^{2}$$

As can be seen, *S_i_* ≤ *S*, and each sample has an equal variance of $\sigma^{2}$:

(11) $$E\left[n^{-1}\left(i-1\right)\left(n-i+1\right){\left({\bar{X}}_{i1}\;-{\bar{\;X}}_{i2}\right)}^{2}\right]=\sigma^{2}+n^{-1}\left(i-1\right)\left(n-i+1\right){(E{\bar{X}}_{i1}\;-\;E{\bar{X}}_{i2})}^{2}$$

If there is no change-point, all the samples have the same expectation, and the second item on the right side of Equation (11) is 0. If there is a change-point, this term is generally non-zero. Therefore, the change-point increases the gap between *S* and *S_i_*. The minimum value of *S*_2_, ⋯, *S_n_* is denoted as S*

(12) $$S^{*}{=\;\min(S}_{2},\cdots {,S}_{n})$$

Moreover, the following test method can be used: when

(13) $${S\;-\;S}^{*}\;>\;C$$

the null hypothesis H is rejected; that is, the original change-point is identified. Otherwise, H is accepted. *C* is an appropriately defined limit, which is determined by

(14) $$\underset{n\to \infty }{\lim}p\left[\frac{{S\;-\;S}^{*}}{\sigma^{2}}\;<\;2\mathrm{lglg}n\;+\;\mathrm{lglglg}n-\;\mathrm{lg}\pi \;+\;x\right]=\;\exp\left({-2e}^{-x/2}\right)$$

If $\sigma^{2}$ is known, for a given level α>0,

(15) $$exp\left(-2e^{-x/2}\right)=1-\alpha$$

(16) $$x_{\alpha }=\;-2\mathrm{lg}(-\frac{1}{2}\mathrm{lg}(1\;-\;\alpha ))$$

(17) $${C\;=\;\sigma }^{2}{(2\mathrm{lglg}n\;+\;\mathrm{lglglg}n\;-\;\mathrm{lg}\pi \;+\;x}_{\alpha })$$

If $\sigma^{2}$ is unknown,

(18) $${\;\sigma }^{2}{\;=\;S}^{*}/(n\;-\;2\mathrm{lglg}n\;-\;\mathrm{lglglg}n\;-\;2.4)$$

#### 2.2.5. Measurement of the Change-Point Magnitude

In Equation (2), *b_k_*_+1_ − *b_k_* is known as the jump degree at change-point *m_k_*, which can be used as a measure of the change-point magnitude. If there is *q* change points, the estimated values are ${\hat{m}}_{1}\cdots {\hat{m}}_{q}$ from small to large, and then the jump degree at the *i* change-point is estimated by

(19) $$\hat{\theta }=\overset{{\hat{m}}_{i+1}-1}{\underset{j=m_{i}}{\sum }}x_{j}/({\hat{m}}_{i+1}-{\hat{m}}_{i})-\overset{{\hat{m}}_{i}}{\underset{j={\hat{m}}_{i-1}}{\sum }}x_{j}/({\hat{m}}_{i}-{\hat{m}}_{i-1})$$

### 2.3. Data Collection

#### 2.3.1. Participants

In this study, we chose female drivers for analysis, because compared to men, women are more likely to experience anxiety [38,39] and get involved in road traffic accidents [40,41]. A total of 36 female novice extroversion drivers were selected to collect eye movement data. Subject driving propensity was determined using the driving propensity questionnaire developed by Wang et al. [42]. Table 1 presents drivers’ driving propensity and their behavior. If a subject drove more than 10,000 kilometers, he would be defined as an experienced driver, and a novice driver otherwise [43]. Participants were aged between 21 and 45 years, and their driving age ranged from 1 to 23 years. In addition, all the subjects had normal hearing, vision (or corrected vision), and color vision.

#### 2.3.2. Experimental Material and Equipment

• Emotion-induction materials

**Figure 2:**
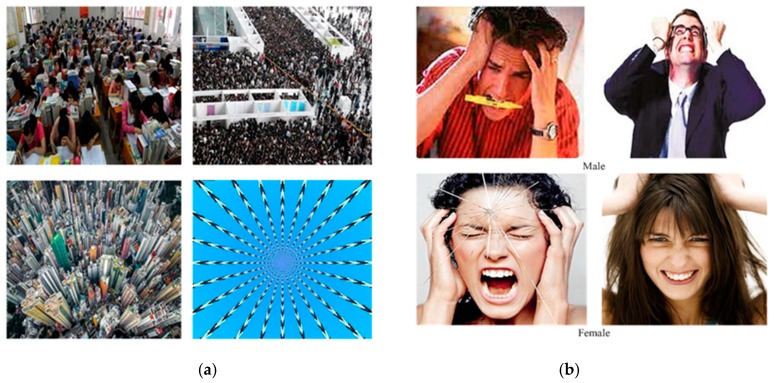
Parts of the anxiety-induction material. (**a**) Visual stimulus; (**b**) Faces of anxiety.

The International Affective Picture System (IAPS) and the Chinese Affective Picture System (CAPS) were used as emotional induction materials. The IAPS is an emotional tool which is generally acknowledged around the world, and the CAPS is an emotional instrument that adapts to the social and cultural context of China. Various anxiety-inducing materials were used in the experiments, including visual materials (e.g., words and pictures, light variation in driving environment), auditory materials (e.g., noisy and irregular sounds), multi-channel materials (e.g., videos and movies), olfactory materials (e.g., cigarettes and durian), and taste materials (e.g., balsam gourd and licorice). Parts of the anxiety induction material are shown in Figure 2.

• Experimental equipment

**Figure 3:**
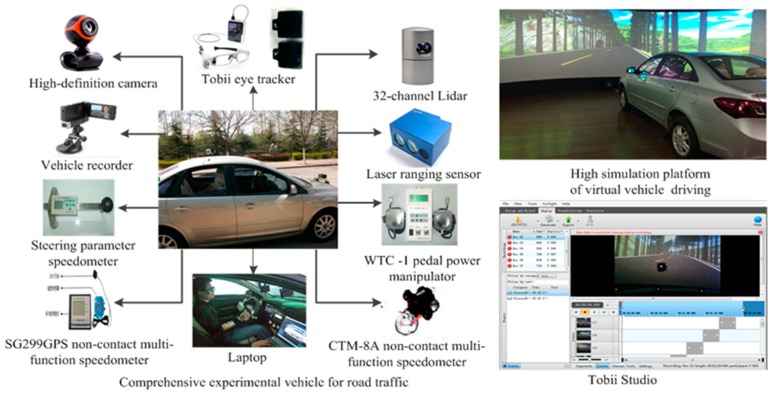
Parts of the experiment instruments.

The experimental equipment includes a comprehensive experimental vehicle (equipped with 32-channel Lidar, laser distance sensor, SG299GPS non-contact multi-function speedometer, CTM-8A non-contact multi-function speedometer, vehicle recorder, Tobii eye tracker, WTC-1 pedal power manipulator, high-definition camera, and laptop, etc.), a high-fidelity driving simulation platform, camera, Tobii Studio software, recorder, IR-Marker, and wedge foam, etc. Parts of the experimental equipment are shown in Figure 3 and Appendix A Table A1.

• Driving route

**Figure 4:**
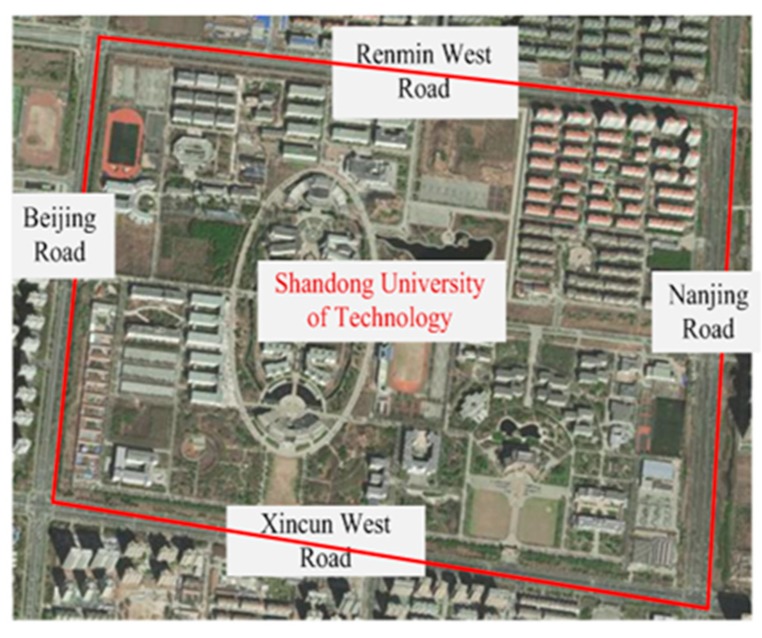
Driving route of the real vehicle experiment.

**Figure 5:**
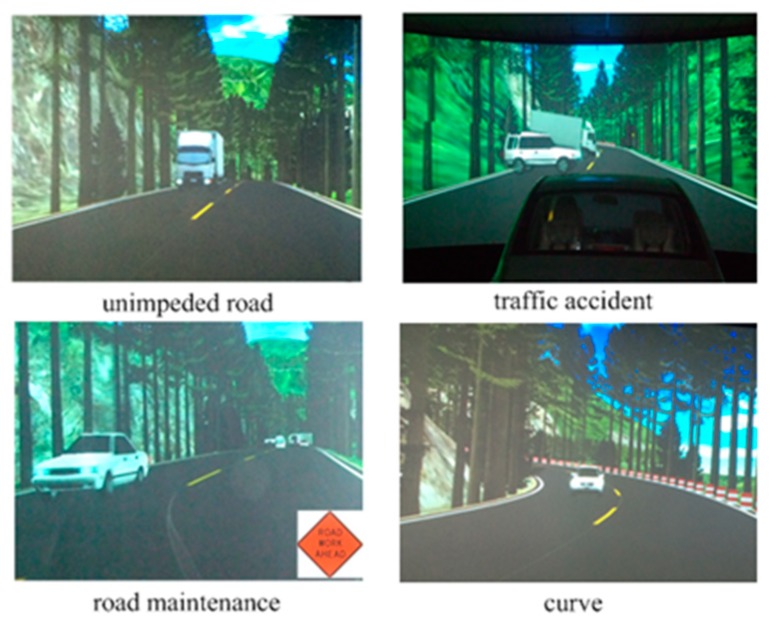
Parts of virtual driving scenes.

The selected route includes Beijing Road, Renmin West Road, Nanjing Road, and Xincun West Road in Zibo city (as shown in Figure 4, total length of 5.6 km). The real driving experiments were conducted on sunny days and favorable road conditions. All the subjects were novice drivers. Considering that it is difficult for drivers to maintain calm state in high-traffic-volume roads, the driving experiments in calm were carried out during the off-peak time on Saturday and Sunday morning. Meanwhile, driving experiments under anxiety were conducted at the early peak and late peak from Monday to Friday. In the driving simulations, a two-lane one-way road with various scenarios was designed (as shown in Figure 5), including unimpeded road, road maintenance, traffic accident, and a curve. A set of pre-defined obstacles on the roads were designed to maintain or increase drivers’ anxiety level during driving.

#### 2.3.3. Experimental Procedure

The driving experiments include real vehicle experiment and driving simulation. Using real driving experiments to collect data is time-consuming, expensive, and difficult to organize. Therefore, it is difficult to obtain a large amount of real driving experimental data. Driving simulation can be used as a supplement to real vehicle experiment, because it is safety, low-cost, and easy to control. Each subject was involved in one real driving experiment and one driving simulation driving experiment on different days. All the experiments were completed in one month.

• Preparation

**Figure 6:**
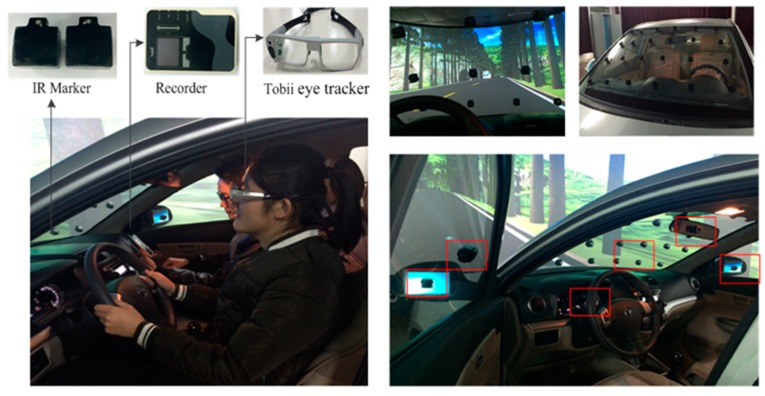
The IR-Marker distribution.

Before each experiment, the IR-Markers of Tobii eye tracker were fixed on the front windshield by a 4 × 6 matrix, and were also placed on the rearview mirror, steering wheel, and dashboard, in accordance with the requirements. The IR-Marker distribution is shown in Figure 6. It can be noted that the IR-Markers were placed to the left since drivers sat on the left side of vehicle. The wedge foam was used to be an offset to the tilt angle of the front windshield in order to ensure that drivers were directly in front of the IR-Markers. Researchers calibrated the eye tracker for each driver strictly before experiment, to collect high-quality and accurate data.

• Emotion induction and driving experiments

**Figure 7:**
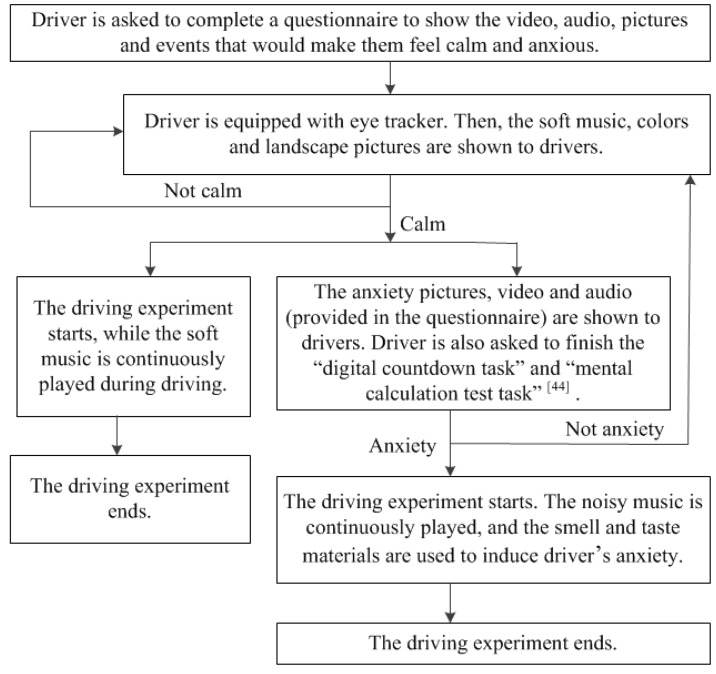
Driving experiment procedure [44].

Anxiety is generally divided into two types: state anxiety and trait anxiety. State anxiety is a temporary emotional response to exogenous stimuli. Trait anxiety is a relatively stable personality feature, which has nothing to do with external stimuli. Anxiety in this study includes drivers’ state anxiety induced in the experiments, and their own trait anxiety. The driving experiment process involving calmness and anxiety is shown in Figure 7.

• Assessing the level of induced anxiety

It is necessary to assess if subjects’ anxiety is induced to a certain level of arousal, because too little or too much arousal can adversely affect subjects’ performance in experiments. During the driving experiments, the facial expression, action, road conditions, driving speed, and pedal strength were recorded in real time with the video monitoring system, speedometer, and pedal dynamics instrument. Subjects were asked to describe their self-perception of emotion in the conversation with experimenters. After the driving experiment, each subject was asked to watch the video immediately and report his emotional experience. The data segments of subject’s anxiety were determined through the Beck Anxiety Inventory, the Self-Rating Anxiety Scale, and subjects’ physiological characteristics such as facial expressions, voice signals, and behavioral actions. The selected data segments were used for the subsequent process and analysis. Table 2 shows the level of anxiety on the Beck Anxiety Inventory and Self-Rating Anxiety Scale. The anxiety induction was considered successful if subjects had a score of 26 points or more on the Beck Anxiety Inventory, and a score of 60 points or more on the Self-Rating Anxiety Scale for anxiety symptoms [45,46]. Therefore, only moderate and severe anxiety were selected for analysis in this study.

### 2.4. Eye Movement Feature Extraction

Subjects were involved in the experiments, in which they were induced to feel either calm or anxious. The experimental data were divided into segments of 100 s each, and a total of 864 effective segments were obtained. Parts of data segments are shown in Table 3.

In this paper, statistical analysis was performed using SPSS Statistics 23.0 (IBM, NY, USA) where the confidence interval was set at 95%. The paired *t*-test was used to determine whether there is a difference between calm and anxiety in driver’s eye movement characteristics. The results are demonstrated in Table 4.

The results show that there is a significant difference in fixation count and fixation duration between states of calmness and anxiety (*p* < 0.05). Drivers have a lower fixation count and a longer fixation duration in a state of anxiety as compared to calmness. Therefore, the two indicators were selected as input parameters in the model, which were used to analyze drivers’ eye movement characteristics in a state of anxiety.

### 2.5. Data Selection and Analysis

**Figure 8:**
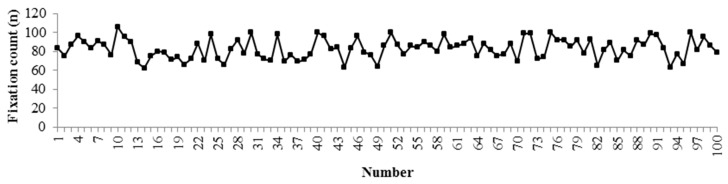
Drivers’ fixation count in anxiety.

**Figure 9:**
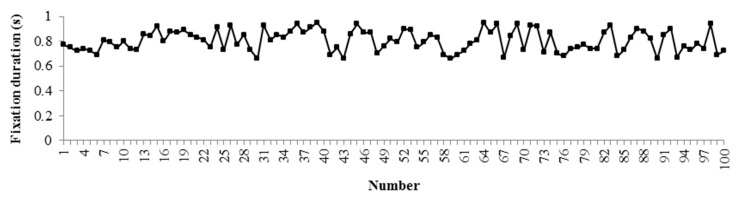
Drivers’ fixation duration in anxiety.

To simplify analysis, only 100 segments of the drivers’ area of interest from the front window were selected and analyzed in this paper. The fixation count and fixation duration for each segment are shown in Figure 8 and Figure 9.

**Figure 10:**
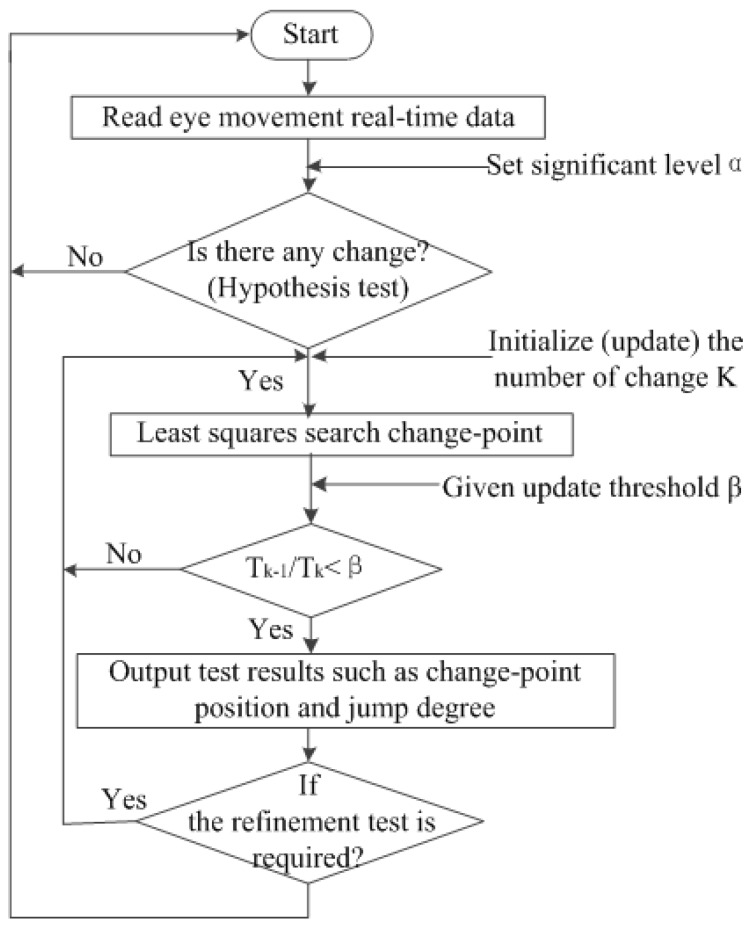
The flow of least squares algorithm.

The least squares method of change-point analysis was used to quickly detect the time and locations of change points to greatly reduce the effects of random interference on eye movement characteristics, following the process shown in Figure 10.

## 3. Results and Discussion

Table 5 and Table 6 show the analysis results of the fixation count and fixation duration using the least squares method. Because the algorithm yields a strong sensitivity for eye movement data, a significance level of *α* = 0.001 was selected, and *β* = 1.01 was chosen to control the number of simulation cycles.

**Figure 11:**
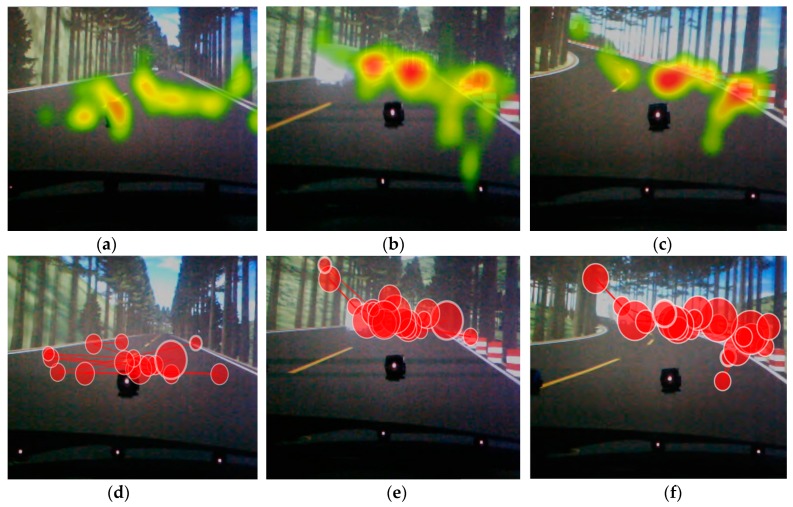
The heat maps and gaze plot maps of drivers’ eye-movement characteristics in anxiety. (**a**) Heat map of fixation count before the change-point occurs; (**b**) Heat map of fixation count before the change-point occurs; (**c**) Heat map of fixation count after change-point 2 occurs; (**d**) Trajectory map of fixation duration before the change-point occurs; (**e**) Trajectory map of fixation duration after change-point 1 occurs; (**f**) Trajectory map of fixation duration after change-point 2 occurs.

Combined with the heat map and gaze plot map of Tobii Studio (shown in Figure 11), drivers’ eye movement data in anxiety were analyzed. Heat maps use different colors to show participants’ attention areas in an image. Using colors on a scale from red to green, heat maps show the highest number of gazes in red. Gaze plot maps show drivers’ eye movements towards the area of interest, where the size of dot represents the duration of a fixation.

The least squares method was used to identify the change points, considering the features of fixation count and fixation duration together. Big changes in eye-movement were found in the 10th, 59th, 65th, and 67th segments. To prevent premature convergence, *β* was calibrated as 1.01 and the algorithm was repeatedly run. Finally, it was determined that the change points occurred in the 10th and 65th segments, where the changes in both fixation count and fixation duration are significant.

Combining with the heat map and gaze plot map, it was found that greater fixation count, higher fixation duration, and greater attention bias appear in the 10th and 65th segments than in others. This might be attributed to the changes in road environment. In the 10th segment, a road traffic accident occurred. This made drivers feel more anxious and pay more attention to the accident scene, which resulted in more gaze points and longer fixation duration. There was a sharp curve in the 65th segment. Drivers had to pay more attention to the curved road, and hold their gaze for longer periods of time. These findings are consistent with the results obtained by the above algorithm.

## 4. Limitations

Our findings of this study suggest that driver’s eye-movement features can be used to detect unexpected events in road environment and improve the geometric design of curved roads. It can also be used to develop active driving warning systems and intelligent human–machine interactions in vehicles. This study would be of great theoretical significance and application value for improving road traffic safety. Further studies are required to confirm the effectiveness of the change point method in detecting changes in drivers’ eye movements, using more experimental data. In addition, further studies are also needed to use more eye-movement metrics to explore drivers’ eye movement characteristics.

## 5. Conclusions

This study used various materials to induce drivers’ anxiety, and then conducted real driving experiments and driving simulations to collect drivers’ eye-movement data. We analyzed the differences between calmness and anxiety in drivers’ eye movement characteristics, using the least squares method of change point to detect eye movement changes of drivers with anxiety. The main findings are demonstrated as follows.

(1) There are significant differences between calmness and anxiety in driver’s fixation count and fixation duration. Female drivers have a lower fixation count and a longer fixation duration in anxiety than in calmness.

(2) Female drivers show a greater fixation count, higher fixation duration, and greater attention bias in scenes with traffic accidents or sharp curves, than in others.

(3) The least squares method of change point analysis is effective to detect eye movement changes of female drivers in anxiety.

## Figures and Tables

**Table 1:** Drivers’ driving propensity type and their behavior.

Driving Propensity Type	Performance
Introversion	Steady, prudent, stable attention and difficult to shift, not easy to generate risk-taking motivation, easy to drive at low speed, fear of traffic accidents.
Middle type	Obey the traffic rules strictly, observe thoughtful, consider the complex traffic situation, more calm, self-control.
Extroversion	Sensitive, active, prone to generate risk-taking motive, rapid response, impetuous in the case of complex traffic, not careful observation.

**Table 2:** The levels of anxiety in the Baker Anxiety Inventory and Self-rating Anxiety Scale.

	Anxiety Level	No Anxiety	Mild Anxiety	Moderate Anxiety	Severe Anxiety
Inventory					
Beck Anxiety Scale		<15	15–25	26–35	>35
Self-Rating Anxiety Scale		<50	50–59	60–69	>69

**Table 3:** Parts of data segments.

Number	Emotion	Fixation Count (*n*)	Fixation Duration (s)	Visit Duration (s)
1	calmness	70	0.66	66.02
	anxiety	60	0.92	89.87
2	calmness	85	0.63	72.81
	anxiety	61	0.88	89.23
…	…	…	…	…
*n* − 1	calmness	87	0.69	74.26
	anxiety	66	0.96	85.00
*n*	calmness	85	0.63	66.64
	anxiety	70	0.82	88.53

**Table 4:** Paired sample *t*-test for drivers’ eye movement characteristics.

Eye Movement Index	Emotion	Mean	Standard Deviation	Correlation Coefficient	*t*	*p*
Fixation count	Calmness	8.44	6.336	0.968	1.933	0.000
	Anxiety	7.00	7.730			
Fixation duration	Calmness	0.66	0.174	0.688	−5.044	0.001
	Anxiety	0.92	0.214			
Visit duration	Calmness	7.34	5.97	0.932	−2.015	0.079
	Anxiety	9.99	8.86			

**Table 5:** The hypothesis test results by the least squares method.

Index	Sig. Level *α*	Variance S	Variance S*	Test Threshold C	S − S* > C
Fixation count	0.001	11,286.24	10,475.24	770.72	Y
Fixation duration	0.001	0.7242	0.6659	0.0486	Y

S: Variance; $S^{*}:\;\mathrm{Variance}\;\left(\mathrm{minimum}\;\mathrm{value}\right);\;C:\;\mathrm{Threshold}\;\mathrm{value};\;$ Y: Yes.

**Table 6:** The change-point search results by the least squares method.

Index	Total Number of Change K	Initial Change Position	Actual Change Position	Jump Degree	T (m_1_,⋯, m_k_) (T_k_)	*β* = 1.01
						T_k-1_/T_k_
Fixation count	1	19	10	97	10,024	
	2	7	10	102	7039	1.424
		75	65	−27		
	3	4	10	99	5746	1.225
		46	40	16		
		82	59	−29		
	4	4	10	104	5717	1.005
		26	24	−15		
		40	51	18		
		77	65	−30		
Fixation duration	1	18	10	0.64	0.5201	
	2	7	10	0.67	0.2749	1.892
		75	67	0.23		
	3	6	10	0.80	0.1958	1.404
		45	24	0.12		
		82	65	0.22		
	4	4	10	0.79	0.1952	1.003
		26	31	0.16		
		41	43	−0.18		
		83	65	0.23		

## Appendix A

**Table A1:** Company information for the experimental equipment.

Equipment	Company	City, Country
High-definition camera	Shenzhen Dishijia Technology Co., Ltd.	Shenzhen, China
Vehicle recorder	Shenzhen Dazhi Innovation Technology Co., Ltd.	Shenzhen, China
Steering parameter speedometer	Zibo Xiangan Electronic Technology Co., Ltd.	Zibo, China
SG299GPS non-contact multi-function speedometer	Beijing Haifuda Technology Co., Ltd.	Beijing, China
CTM-8A non-contact multi-function speedometer	Zibo Aoke Electronics Co., Ltd.	Zibo, China
Laptop	Lenovo Group Limited	Beijing, China
WTC-1 pedal power manipulator	Zibo Chuangyu Electronics Co., Ltd.	Zibo, China
Laser distance sensor	Wuhan Jiguangsheng Measurement and Control Co., Ltd.	Wuhan, China
32-channel Lidar	Beijing Dongrong Shengshi Technology Co., Ltd.	Beijing, China
Tobii eye tracker	Beijing Jinfa Technology Co., Ltd.	Sweden
